# The role of peripheral blood HIF-1α in pancreatic β-cell dysfunction and insulin resistance among patients with type 2 diabetes: a systematic review and meta-analysis

**DOI:** 10.3389/fnut.2026.1763090

**Published:** 2026-04-10

**Authors:** Yi Su, Rui Peng, Ting Luo, Qingzhi Liang, Jiahong Zhang, Xianglong Li, Yulin Leng, Xiaoxu Fu, Chunguang Xie

**Affiliations:** 1 Hospital of Chengdu University of Traditional Chinese Medicine, Chengdu, Sichuan, China; 2 TCM Prevention and Treatment of Metabolic and Chronic Diseases Key Laboratory of Sichuan Province, Hospital of Chengdu University of Traditional Chinese Medicine, Chengdu, Sichuan, China; 3 Department of Endocrinology, Hospital of Chengdu University of Traditional Chinese Medicine, Chengdu, Sichuan, China

**Keywords:** hypoxia-inducible factor-1α, insulin resistance, meta-analysis, pancreatic β-cell function, type 2 diabetes mellitus

## Abstract

**Importance:**

Elucidating the role of hypoxia-inducible factor-1α (HIF-1α) in pancreatic β-cell dysfunction is essential for understanding the pathophysiology of type 2 diabetes mellitus (T2DM) and improving clinical risk assessment.

**Objective:**

To evaluate associations between protein expression level of HIF-1α in peripheral blood and key glycemic parameters—fasting plasma glucose (FPG), glycated hemoglobin (HbA1c), fasting insulin (FINS), and the homeostatic model assessment of insulin resistance (HOMA-IR)—in patients with T2DM.

**Data sources:**

PubMed, Embase, Cochrane Library, Web of Science (WOS), Wanfang, China National Knowledge Infrastructure (CNKI), China Science and Technology Journal Database (VIP), and Chinese Biomedical Literature Service System (SinoMed) were searched from inception to July 28, 2025, for Chinese and English publications.

**Study selection:**

Observational studies examining the relationship between HIF-1α levels and glycemic outcomes in T2DM populations.

**Data extraction and synthesis:**

Two reviewers independently extracted data and assessed study quality using AHRQ criteria. Random-effects meta-analysis was used to calculate mean differences (MD) with 95% confidence intervals (CI).

**Main outcomes and measures:**

Differences in FPG, HbA1c, FINS, and HOMA-IR between high and low HIF-1α groups with T2DM.

**Results:**

Thirty-six studies (*n* = 5,979) were included. Elevated peripheral HIF-1α was significantly associated with increased FPG (MD = 1.13, 95% CI [0.59, 1.67]), HbA1c (MD = 0.93, 95% CI [0.63, 1.24]), FINS (MD = 1.13, 95% CI [0.24, 2.02]), and HOMA-IR (MD = 1.40, 95% CI [0.62, 2.18]). Subgroup analysis indicated that geographic location significantly modified the FPG association (*p* = 0.0105). Sensitivity analyses confirmed the robustness of these findings.

**Conclusion:**

Elevated peripheral HIF-1α levels correlate with impaired β-cell function and increased insulin resistance. The discordance between elevated systemic levels and known tissue-specific reductions suggests that peripheral HIF-1α may reflect a compensatory response to hypoxia rather than a primary driver of T2DM pathogenesis.

**Systematic review registration:**

https://www.crd.york.ac.uk/PROSPERO/view/CRD420251118501, CRD420251118501.

## 1 Introduction

T2DM represents a major global public health challenge, imposing a substantial and increasing burden on healthcare systems. Estimates from 2021 indicate that approximately 529 million individuals were living with diabetes—an age-standardized global prevalence of 6.1%—with T2DM accounting for roughly 96% of cases (1). This prevalence is projected to reach 12.2% by 2045, potentially affecting 783.2 million people. The pathophysiology of T2DM involves both pancreatic β-cell dysfunction and insulin resistance (2). Notably, the UK Prospective Diabetes Study reported that β-cell function is often reduced to 50% of normal capacity by the time of diagnosis and declines to approximately 28% within 6 years (3). This progressive deterioration drives worsening glycemic control and poor treatment response, while chronic hyperglycemia and glucose fluctuations significantly increase the risk of diabetes-related complications (4, 5). Therefore, elucidating the molecular mechanisms regulating β-cell function and insulin resistance is essential for developing strategies to halt T2DM progression.

Beyond its established role as a central regulator of cellular responses to hypoxia, HIF-1α has gained significant attention in metabolic research. Clinical studies have reported aberrant HIF-1α expression in the peripheral blood of T2DM patients, and genetic evidence from Japanese cohorts has established a correlation between HIF-1α polymorphisms and T2DM susceptibility, implicating the factor in glucose homeostasis and islet function (6–8). Mechanistic studies further suggest that HIF-1α contributes to β-cell dysfunction and insulin resistance by modulating pathways related to insulin signaling, oxidative stress, cellular dedifferentiation, and autophagy. Collectively, these findings identify HIF-1α as a pivotal molecular node in T2DM pathogenesis (9–11). Nevertheless, a systematic synthesis of clinical evidence quantifying the association between circulating HIF-1α levels and markers of β-cell function and insulin resistance remains unavailable.

While several studies have investigated the relationship between peripheral HIF-1α protein levels and β-cell function, their conclusions remain inconsistent. This is likely due to limitations such as small sample sizes, heterogeneous study designs, and restricted population demographics. Furthermore, a comprehensive quantitative synthesis of the correlation between HIF-1α and key clinical measures, such as FPG, HbA1c, FINS, and the HOMA-IR, is currently lacking.

To address this gap, we conducted a systematic review and meta-analysis adhering to evidence-based medicine principles. By pooling data from available studies, this research aims to clarify the correlation between peripheral HIF-1α expression and key indicators of pancreatic function and insulin resistance. Furthermore, we employed subgroup analyses and meta-regression to investigate potential sources of heterogeneity, aiming to provide a robust foundation for clinical practice and future research. By rigorously evaluating the relationship between HIF-1α and metabolic parameters (FPG, HbA1c, FINS, and HOMA-IR), this study contributes evidence to support the potential role of HIF-1α as both a metabolic assessment biomarker and a therapeutic target in T2DM.

## 2 Methods

This systematic review and meta-analysis was conducted in accordance with PRISMA 2020 guidelines (Supplementary file S1). The study protocol was prospectively registered with PROSPERO (CRD420251118501, Supplementary file S2).

### 2.1 Search strategy

A systematic literature search was performed across multiple electronic databases, including PubMed, Embase, the Cochrane Library, Web of Science (WOS), Wanfang, CNKI, VIP, and SinoMed, from inception through July 28, 2025. We utilized a combination of MeSH terms and free-text keywords to identify relevant studies in both English and Chinese. Search terms included “type 2 diabetes mellitus” “hypoxia-inducible factor 1, alpha subunit,” and their variations. The full search strategy for each database is available in Supplementary file S3.

### 2.2 Eligibility criteria

Studies were included based on the following criteria: (1) Population: Adult patients diagnosed with T2DM, regardless of disease duration. (2) Exposure: The protein expression level of HIF-1α in peripheral blood, represented by serum or plasma HIF-1α concentration measured by ELISA. (3) Comparison: Variation in pancreatic islet function and insulin resistance parameters according to peripheral blood HIF-1α protein expression level. (4) Outcome measures: Primary outcomes included FPG, HbA1c, FINS, and HOMA-IR. (5) Study design: Observational studies, including cross-sectional, case–control, and cohort designs.

### 2.3 Exclusion criteria

Studies were excluded for any of the following: (1) Non-original research publications (e.g., reviews, meta-analyses, editorials, conference abstracts). (2) Populations not exclusively with T2DM. (3) Lack of simultaneous measurement of peripheral blood HIF-1α protein expression level and relevant pancreatic β-cell function or insulin resistance indices. (4) Single-arm studies without a comparative control or where effect estimates could not be derived. (5) Unavailability of data even after contacting the corresponding authors.

### 2.4 Study selection and data extraction

Two investigators independently screened studies using EndNote 20. Following duplicate removal, titles, abstracts, and full texts were reviewed against the eligibility criteria. Disagreements were resolved through consensus or consultation with a third reviewer. A standardized form was used to extract: lead author, country, sample size, participant demographics (age, sex), disease duration, study design, sample type, assay method, HIF-1α values, and outcome data (FPG, HbA1c, FINS, HOMA-IR). For studies reporting results as medians and ranges, means and standard deviations were estimated using established methods (12).

### 2.5 Assessment of methodological quality

Methodological quality was independently assessed by two investigators using the Agency for Healthcare Research and Quality (AHRQ) criteria for cross-sectional studies. This 11-item tool categorizes study quality as low (0–3), intermediate (4–7), or high (8–11). Discrepancies were resolved via discussion or third-party arbitration.

### 2.6 Statistical synthesis and analysis

Quantitative synthesis was performed using the meta package in R (version 4.5.2). Continuous outcomes were synthesized as mean differences (MD) with 95% confidence intervals (CI) using a random-effects model. Heterogeneity was quantified using the *I*^2^ statistic; *I*^2^ ≤ 50% indicated low heterogeneity (fixed-effects model), while *I*^2^ > 50% indicated substantial heterogeneity (random-effects model) (13). Subgroup analyses, stratified by country, and Chinese region, were implemented via the byvar parameter to explore potential sources of inconsistency. The robustness of the outcomes was evaluated through leave-one-out sensitivity analyses using the metainf function. Draw a funnel plot using the funnel function and evaluate publication bias using the Egger test combined with the metabias function. Furthermore, model diagnostics were performed by extracting random-effects residuals for Q-Q plot visualization, with the normality assumption verified by Shapiro–Wilk tests. To further investigate heterogeneity, meta-regression based on restricted maximum likelihood (REML) estimation was conducted for age and disease duration using the metareg command in Stata 18.0. All statistical tests were two-sided, with significance set at α = 0.05. These analyses strictly adhered to standard reporting guidelines to ensure the validity and reproducibility of the results.

## 3 Results

### 3.1 Literature search and study selection

**Figure 1:**
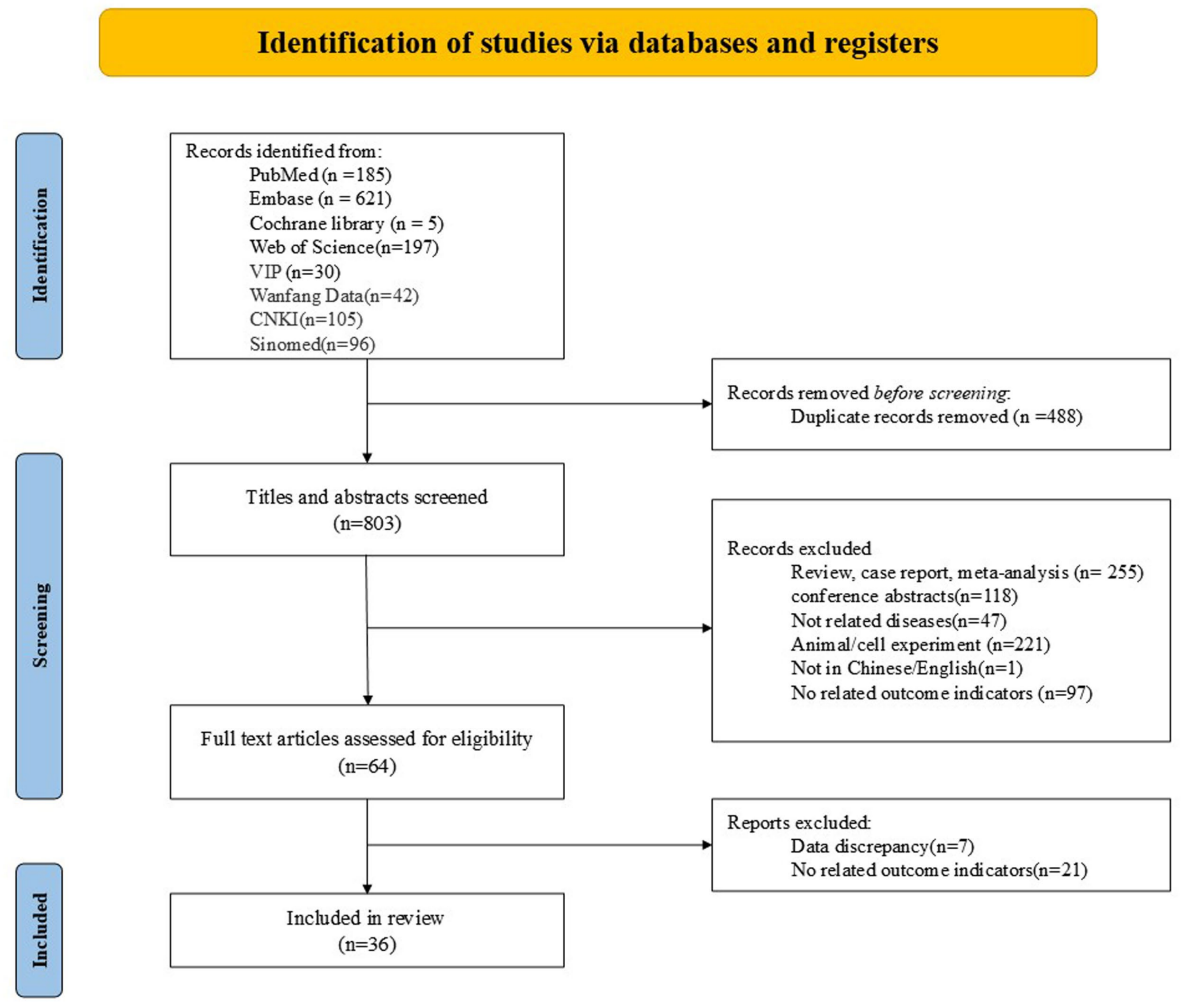
Literature screening flowchart.

The initial search yielded 1,291 candidate articles. After removing 488 duplicates, 803 articles were screened by title and abstract, 64 of which qualified for full-text review. Following a detailed assessment, 28 articles were excluded (with reasons provided in Figure 1). Ultimately, 36 studies met all inclusion criteria for quantitative synthesis. The selection process is detailed in the PRISMA flowchart (Figure 1).

### 3.2 Characteristics and quality of included studies

The 36 included studies were all cross-sectional, encompassing a total of 5,979 patients with T2DM. Published between 2010 and 2025, the research was primarily conducted in China (30 studies), with additional studies from Indonesia and Saudi Arabia (6 studies). Sample sizes ranged from 36 to 556, and the mean age of participants spanned 44.22–67.58 years. Detailed baseline characteristics are summarized in Table 1.

**Table 1:** Basic characteristics of included studies.

General information				Demographic information of population				Study design	Source	Method	Experience group		Control group		Outcome
First author	Publication year	Country	Sample size	Age	Duration	Gender (M/F)					High_group_N	High_group-HIF-1α (pg/ml)	Low_group_N	Low_group-HIF-1α (pg/ml)	
Shao et al. (29)	2016	China	314	55.49	8.71	148	166	Cross sectional study	Serum	Elisa	133	35.78 ± 3.87	181	23.21 ± 7.08	FPG, HbA1c, FINS, HOMA-IR
Shao et al. (30)	2016	China	333	55.00	9.10	/	/	Cross sectional study	Serum	Elisa	140	36 ± 4	193	24 ± 7	FPG, HbA1c, FINS, HOMA-IR
Che et al. (31)	2023	China	556	49.30	/	306	250	Cross sectional study	Serum	Elisa	276	113.69 ± 18.85	280	72.16 ± 10.33	FPG, HbA1c
Tang et al. (32)	2020	China	108	58.05	9.49	55	53	Cross sectional study	Serum	Elisa	55	635.26 ± 180.56	53	486.23 ± 136.01	FPG, HbA1c
Shen et al. (33)	2021	China	40	/	/	/	/	Cross sectional study	Blood	Elisa	20	(2.67 ± 0.21) × 10^9^	20	(1.98 ± 0.32) × 10^9^	FPG, HbA1c
E and Shi (34)	2012	China	56	56.57	/	31	25	Cross sectional study	Serum	Elisa	32	1,550 ± 710	24	950 ± 450	FPG, HbA1c, FINS, HOMA-IR
Zhang et al. (35)	2023	China	110	57.51	5.04	62	48	Cross sectional study	Serum	Elisa	55	39.83 ± 5.51	55	27.74 ± 4.0	FPG, HbA1c, FINS
Liu et al. (36)	2013	China	88	55.95	5.44	50	38	Cross sectional study	Serum	Elisa	43	289 ± 66	45	214 ± 56	FPG, HbA1c
Li et al. (37)	2016	China	92	52.38	/	45	47	Cross sectional study	Plasma	Elisa	35	0.2617 ± 0.0182	57	0.1802 ± 0.0125	FPG, HbA1c
Li et al. (38)	2024	China	200	66.70	16.08	145	55	Cross sectional study	Serum	Elisa	98	335.02 ± 149.72	102	295.35 ± 118.13	FPG, HbA1c
Lu et al. (39)	2021	China	94	59.50	/	47	47	Cross sectional study	Serum	Elisa	49	7,090 ± 820	45	1,280 ± 540	FPG, HbA1c
Lu et al. (40)	2014	China	208	51.63	7.12	128	80	Cross sectional study	Serum	Elisa	102	>209.8	106	<139.8	FPG, HbA1c
Shao et al. (41)	2017	China	278	49.94	9.50	141	137	Cross sectional study	Serum	Elisa	115	33.3 ± 4.0	163	23.7 ± 7.2	FPG, HbA1c, FINS, HOMA-IR
Shao et al. (42)	2016	China	284	54.42	9.03	151	133	Cross sectional study	Serum	Elisa	116	35.61 ± 3.97	168	23.15 ± 7.24	FPG, HbA1c, FINS, HOMA-IR
Song et al. (43)	2016	China	77	50.40	/	/	/	Cross sectional study	Serum	Elisa	34	0.13150 ± 0.03026	43	0.06522 ± 0.01831	HbA1c
Song et al. (44)	2016	China	76	52.59	7.15	41	35	Cross sectional study	Plasma	Elisa	28	0.2530 ± 0.0163	48	0.0703 ± 0.0143	FPG, HbA1c
Sun et al. (45)	2024	China	200	62.69	/	96	104	Cross sectional study	Serum	Elisa	100	900 ± 220	100	640 ± 260	FPG, HbA1c, HOMA-IR
Xu et al. (46)	2018	China	182	/	/	/	/	Cross sectional study	Blood	Elisa	49	95.70 ± 31.10	133	80.20 ± 35.50	FPG, FINS, HOMA-IR
Xu et al. (47)	2015	China	160	56.50	/	87	73	Cross sectional study	Serum	Elisa	80	1,560 ± 780	80	940 ± 410	FPG, HbA1c, FINS
Xue et al. (48)	2021	China	36	56.34	9.32	22	14	Cross sectional study	Serum	Elisa	21	1,210 ± 330	15	660 ± 260	FPG, HbA1c
Zhang et al. (49)	2016	China	53	54.51	/	27	26	Cross sectional study	Serum	Elisa	27	1,520 ± 610	26	910 ± 330	FPG, HbA1c, FINS, HOMA-IR
Zhu et al. (50)	2017	China	319	/	7.91	/	/	Cross sectional study	Serum	Elisa	102	36.02 ± 4.45	217	24.06 ± 7.53	FPG, HbA1c, FINS, HOMA-IR
Shan et al. (51)	2010	China	66	55.00	6.01	/	/	Cross sectional study	Serum	Elisa	30	0.12017 ± 0.03146	36	0.07833 ± 0.02102	FPG, HbA1c
Tian et al. (52)	2020	China	130	44.22	/	77	53	Cross sectional study	Serum	Elisa	68	28.2 ± 4.2	62	25.1 ± 2.9	FPG, 2hPBG
Su et al. (53)	2025	China	70	64.11	5.84	32	38	Cross sectional study	Serum	Elisa	45	2,980 ± 1,319	25	1,560 ± 830	FPG, HbA1c
Shao et al. (54)	2017	China	326	55.48	8.69	168	158	Cross sectional study	Serum	Elisa	140	35.68 ± 3.86	186	23.28 ± 7.14	FPG, HbA1c, FINS, HOMA-IR
Shao et al. (55)	2016	China	331	49.69	8.95	168	163	Cross sectional study	Serum	Elisa	130	33.22 ± 3.89	201	24.38 ± 7.24	FPG, HbA1c, FINS, HOMA-IR
Sayed et al. (56)	2016	Egypt	100	53.00	11.00	39	61	Cross sectional study	Serum	Elisa	50	190.7 ± 5.8	50	147.5 ± 13.5	FPG, HbA1c
Rusdiana et al. (57)	2020	Indonesia	89	58.22	/	/	/	Cross sectional study	Serum	Elisa	40	1,670 ± 590	49	690 ± 120	FPG, HbA1c
Lv et al. (8)	2015	China	205	52.65	/	110	95	Cross sectional study	Serum	Elisa	68	537.22 ± 89.05	137	247.82 ± 40.81	HbA1c, HOMA-IR
Li et al. (7)	2014	China	208	51.63	7.12	128	80	Cross sectional study	Serum	Elisa	102	>209.8	106	<139.8	FPG, HbA1c
Jiang et al. (58)	2013	China	60	60.84	/	/	/	Cross sectional study	Serum	Elisa	40	2448.51 ± 704.53	20	2366.19 ± 549.04	FPG, HbA1c
GHARIB et al. (59)	2022	Saudi Arabia	100	51.80	/	51	49	Cross sectional study	Serum	Elisa	50	96.95 ± 18.58	50	71.39 ± 20.54	FPG, HbA1c
Gharib et al. (60)	2021	Saudi Arabia	118	54.93	/	66	52	Cross sectional study	Serum	Elisa	58	34.77 ± 6.43	60	24.86 ± 4.57	FPG, HbA1c
Gaonkar et al. (61)	2020	India	222	60.20	7.63	/	/	Cross sectional study	Plasma	Elisa	74	2,636 ± 360.26	148	692.25 ± 128.24	FPG, HbA1c, FINS
Cuore et al. (6)	2023	Italy	90	67.58	/	64	26	Cross sectional study	Serum	Elisa	50	40,180 ± 10,800	40	33,500 ± 6,160	HbA1c

All studies quantified peripheral blood HIF-1α protein expression level using ELISA. In the included diabetes population, participants were divided into the experience group (high expression group) and the control group (low expression group) according to the relative HIF-1α expression level. Most studies reported outcomes for FPG, HbA1c, FINS, and HOMA-IR.

Methodological quality, assessed via the AHRQ tool, was high for 28 studies, while 8 were rated as intermediate. Common reasons for quality downgrading included non-consecutive patient recruitment, lack of follow-up, and insufficient documentation regarding the recruitment period or quality control procedures. Additionally, some studies failed to justify patient exclusions or provide protocols for handling missing data. Detailed risk-of-bias assessments are available in Supplementary file S4.

### 3.3 Meta-analysis

#### 3.3.1 Primary outcomes

##### 3.3.1.1 FPG

**Figure 2:**
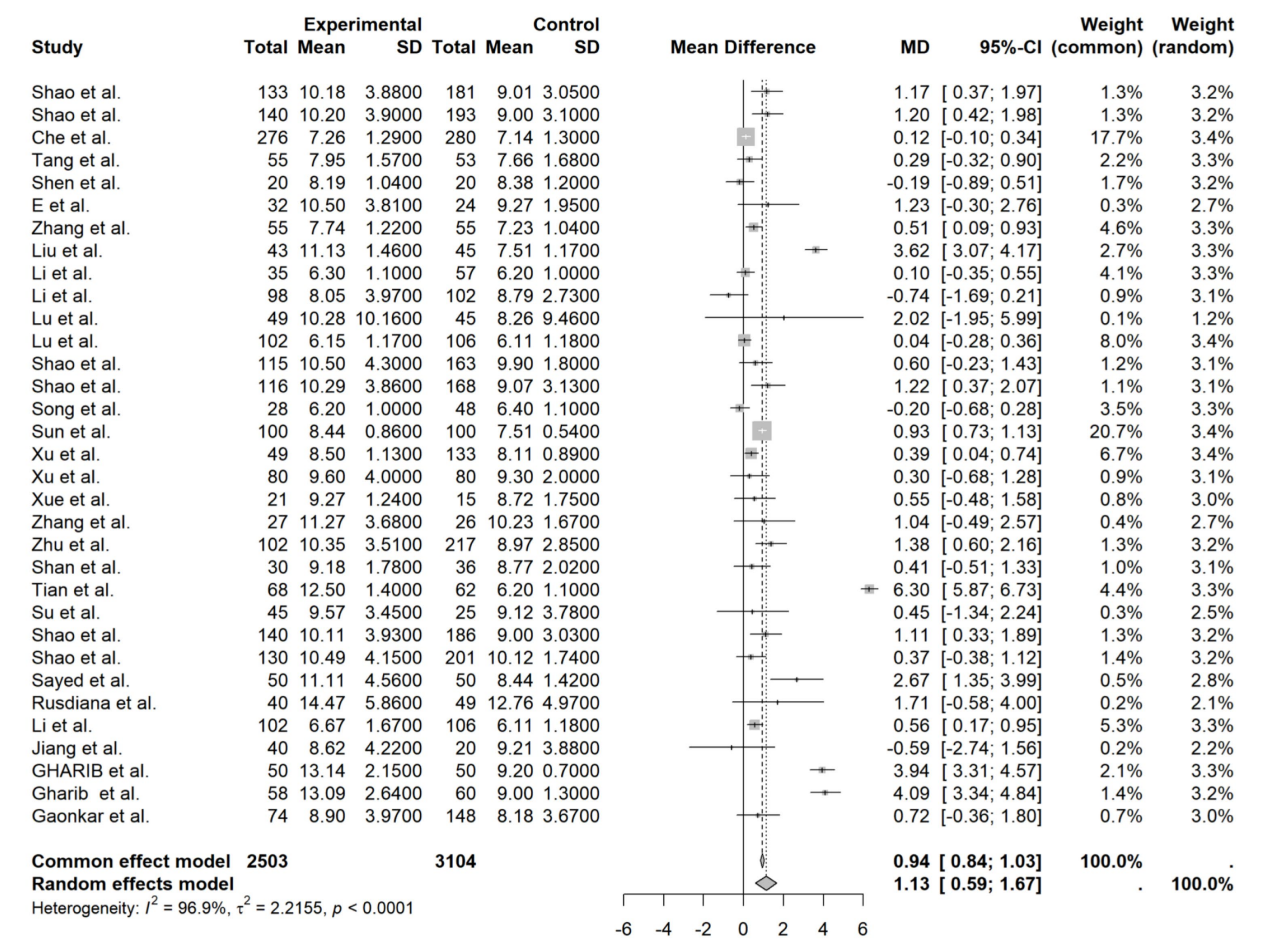
FPG forest plot.

Thirty-three studies reported FPG outcomes. Due to substantial heterogeneity (*I*^2^ = 96.9%, *p* < 0.0001), a random-effects model was employed. The pooled results showed that the high HIF-1α group had significantly higher FPG levels than the low-expression group (MD = 1.13, 95%CI [0.59, 1.67], *p* < 0.0001). This indicates a consistent association between elevated circulating HIF-1α and increased FPG (Figure 2).

##### 3.3.1.2 HbA1c

**Figure 3:**
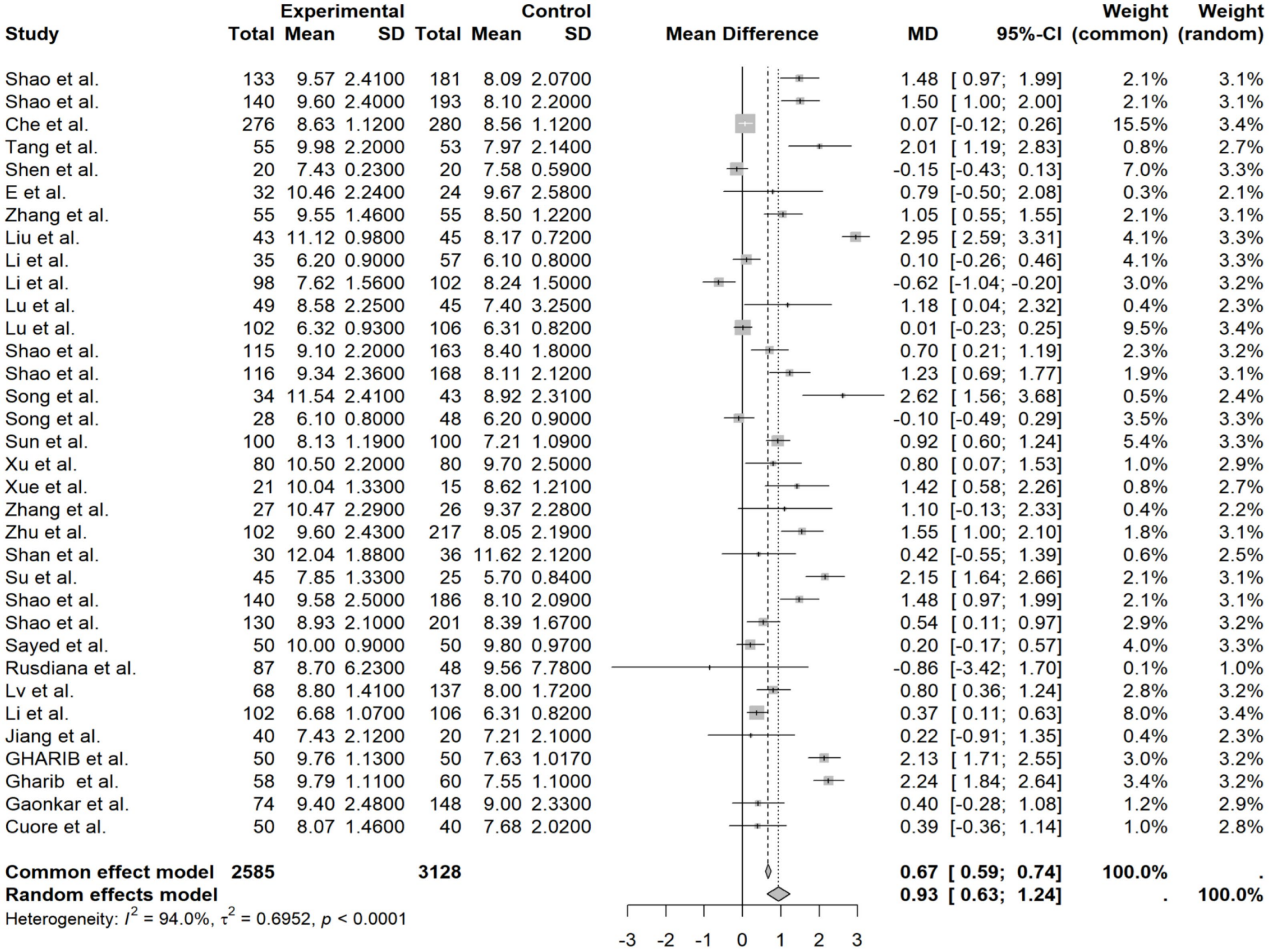
HbA1c forest plot.

Data from 34 studies reporting HbA1c also exhibited high heterogeneity (*I*^2^ = 94.0% *p* < 0.0001). Meta-analysis revealed a significant elevation in HbA1c in the high-expression group (MD = 0.93, 95%CI [0.63, 1.24], *p* < 0.0001), underscoring a robust association between elevated HIF-1α and poorer long-term glycemic control (Figure 3).

##### 3.3.1.3 FINS

**Figure 4:**
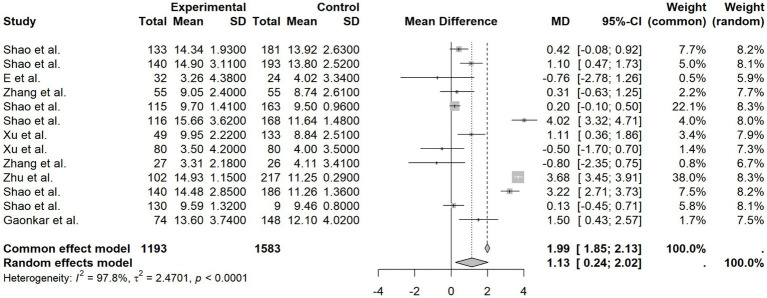
FINS forest plot.

Pooled analysis of 13 studies showed significant differences in FINS levels between groups (*I*^2^ = 97.8%, *p* < 0.0001). Patients with high HIF-1α expression exhibited significantly higher FINS levels compared to the control group (MD = 1.13, 95% CI [0.24, 2.02], *p* < 0.0001), as illustrated in the forest plot (Figure 4). This suggests a consistent association between higher protein expression level of HIF-1α in peripheral blood and increased FINS across the included studies.

##### 3.3.1.4 HOMA-IR

**Figure 5:**
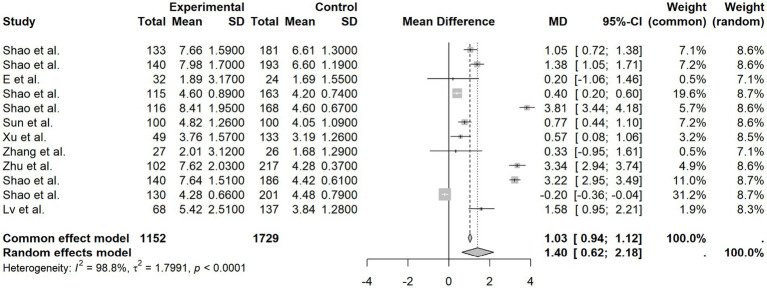
HOMA-IR forest plot.

Twelve studies provided HOMA-IR values. Analysis using a random-effects model (*I*^2^ = 98.8%, *p* < 0.0001) demonstrated that the high-expression group had significantly greater insulin resistance than the control group (MD = 1.40, 95% CI [0.62, 2.18], *p* < 0.0001). These findings suggest a consistent link between increased circulating HIF-1α and exacerbated insulin resistance (Figure 5).

#### 3.3.2 Subgroup analyses

To explore the substantial heterogeneity observed, subgroup analyses were performed using a random-effects model, stratified by age, country, and geographical region within China (North vs. South). The results indicated that geographic location significantly influenced the association between HIF-1α and FPG (*p* = 0.0105). Specifically, the pooled effect size in non-Chinese populations (5 items, MD = 2.74, 95%CI [1.41, 4.07]) was significantly higher than in Chinese populations (28 items, MD = 0.86, 95%CI [0.33, 1.40]). Conversely, regional differences within China (*p* = 0.72) and age stratification (*p* = 0.88) did not significantly alter the associations for FPG, HbA1c, or HOMA-IR. These findings suggest that national or ethnic background may be a primary driver of the observed heterogeneity. Detailed subgroup results are provided in Tables 2–4.

**Table 2:** Subgroup analysis results of FPG.

Item	Subgroup	Number of studies included	Results of heterogeneity test		Meta-analysis results		
			*I*^2^ (%)	*P*	MD	95%CI	*P*
Country	China	28	96.8	<0.0001	0.86	[0.33, 1.40]	0.0105
	Not China	5	87.8	<0.0001	2.74	[1.41, 4.07]	
Region of China	North China	16	97.9	<0.0001	0.95	[0.14, 1.76]	0.7202
	South China	12	92.2	<0.0001	0.76	[0.12, 1.41]	

**Table 3:** Subgroup analysis results of HbA1c.

Item	Subgroup	Number of studies included	Results of heterogeneity test		Meta-analysis results		
			*I*^2^ (%)	*P*	MD	95%CI	*P*
Country	China	28	93.7	<0.0001	0.92	[0.60, 1.25]	0.9658
	Not China	6	94.2	<0.0001	0.95	[0.06, 1.83]	
Region of China	North China	16	89.3	<0.0001	0.84	[0.46, 1.22]	0.6236
	South China	12	96	<0.0001	0.92	[0.44, 1.59]	

**Table 4:** Subgroup analysis results of HOMA-IR.

Item	Subgroup	Number of studies included	Results of heterogeneity test		Meta-analysis results		
			*I*^2^ (%)	*P*	MD	95%CI	*P*
Region of China	North China	9	98.9	<0.0001	1.37	[0.47, 2.26]	0.9109
	South China	3	98.0	<0.0001	1.49	[−0.42, 3.39]	

#### 3.3.3 Meta-regression

**Figure 6:**
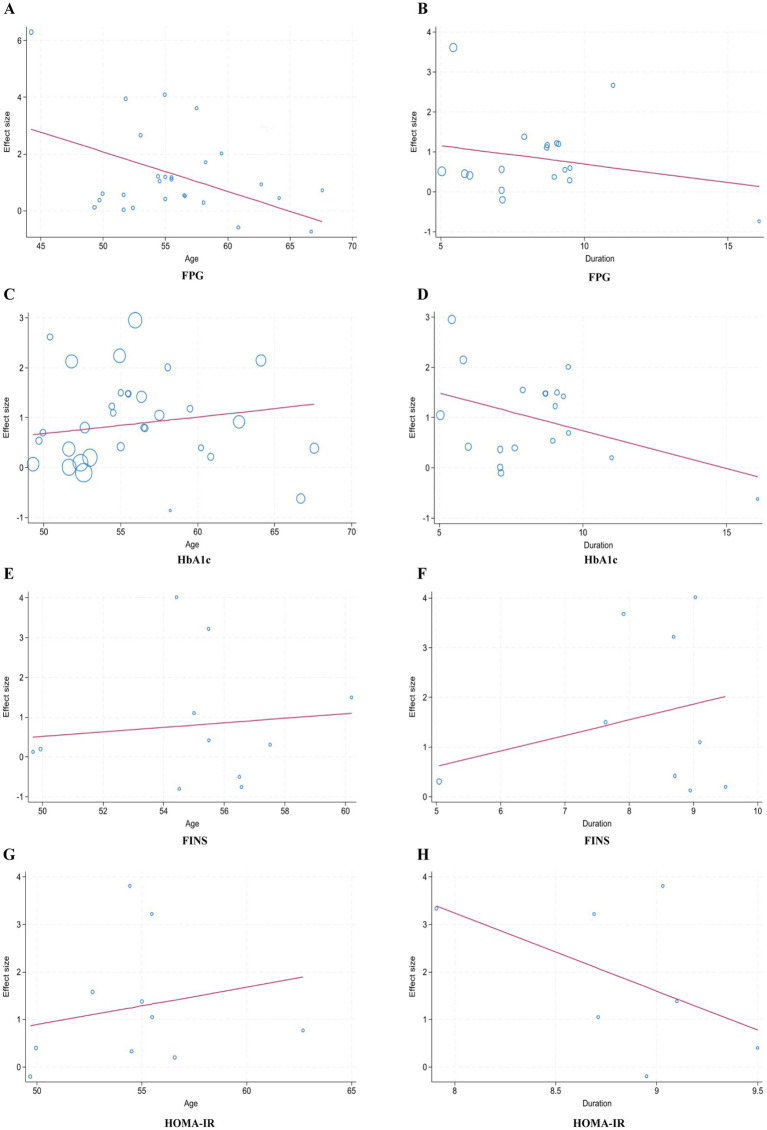
Regression analysis. The horizontal axis represents covariates, and the vertical axis represents the effect size. Each bubble represents an individual study included in the meta-analysis. **(A)** The regression analysis of age and FPG; **(B)** the regression analysis of duration and FPG; **(C)** the regression analysis of age and HbA1c; **(D)** the regression analysis of duration and HbAlc; **(E)** the regression analysis of age and FINS; **(F)** the regression analysis of duration and FINS; **(G)** the regression analysis of age and HOMA-IR; **(H)** the regression analysis of duration and HOMA-IR.

To further explore the observed heterogeneity, meta-regression using the REML method was performed, evaluating the potential influence of age and disease duration. The analysis revealed that neither factor significantly affected the pooled mean differences for FPG, HbA1c, FINS, or HOMA-IR (*p* > 0.05). These findings suggest that the substantial heterogeneity likely stems from individual-level patient variations rather than macro-level study characteristics (Figure 6). Given the remaining residual heterogeneity, future research incorporating individual participant data (IPD) is warranted to better control for potential confounders.

#### 3.3.4 Sensitivity analysis

**Figure 7:**
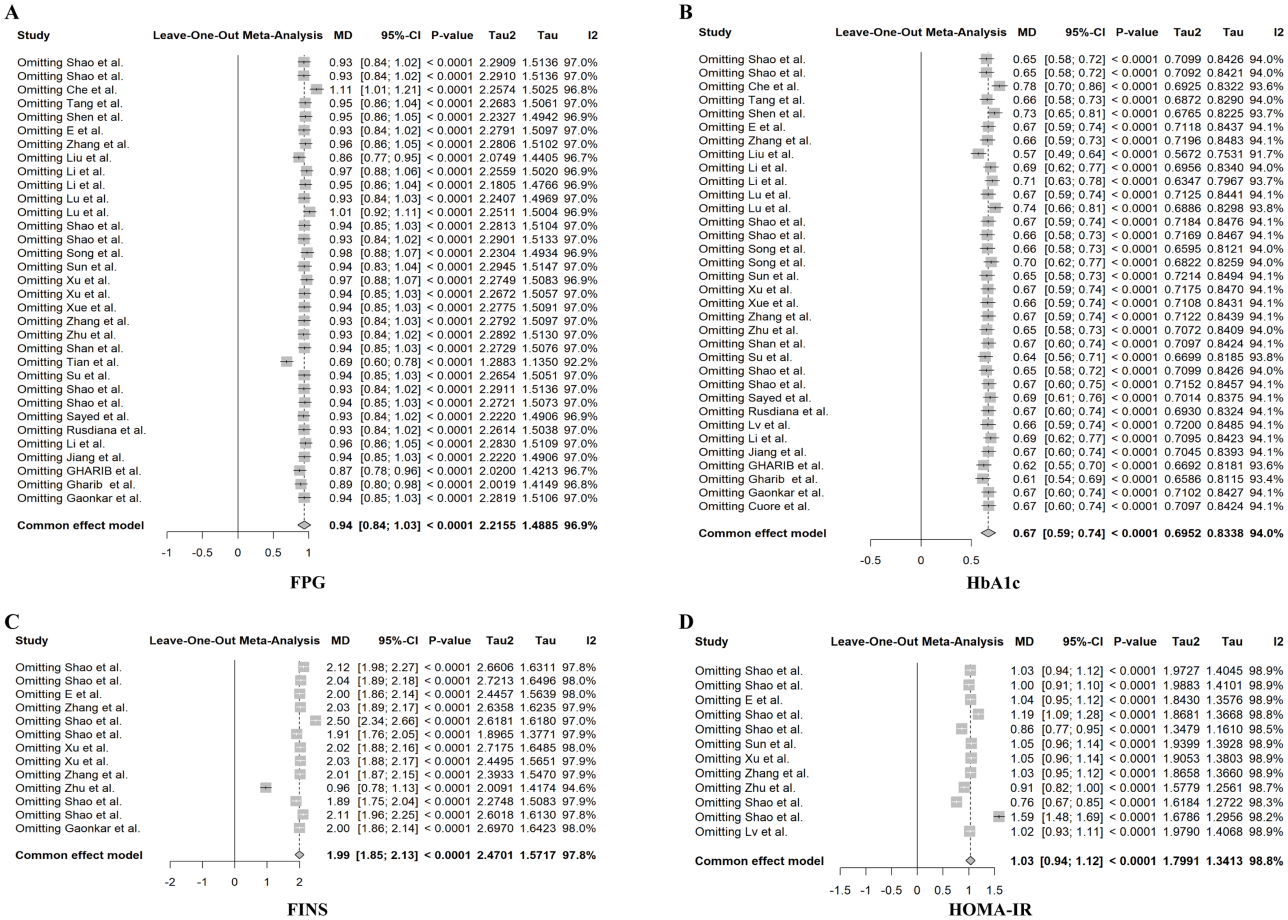
Sensitivity analysis. **(A)** Sensitivity analysis of FPG; **(B)** sensitivity analysis of HbA1c; **(C)** sensitivity analysis of FINS; **(D)** sensitivity analysis of HOMA-IR.

To evaluate the stability of the pooled estimates, we excluded the included studies one by one to evaluate the stability and reliability of the combined results. Systematically excluding individual studies did not yield significant changes in the direction or magnitude of the effect sizes for any outcome. These results confirm the robustness and reliability of our primary findings (Figure 7).

#### 3.3.5 Assessment of publication bias

**Figure 8:**
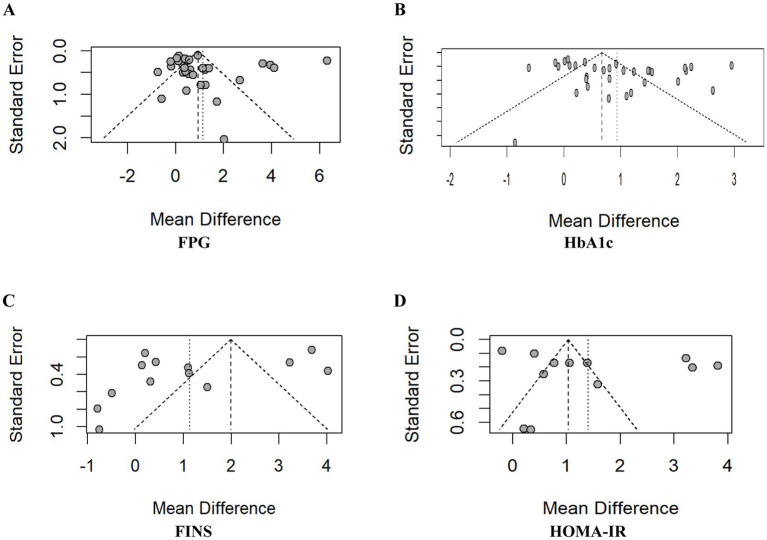
Funnel plot. **(A)** Funnel plot of FPG; **(B)** funnel plot of HbA1c; **(C)** funnel plot of FINS; **(D)** funnel plot of HOMA-IR.

**Figure 9:**
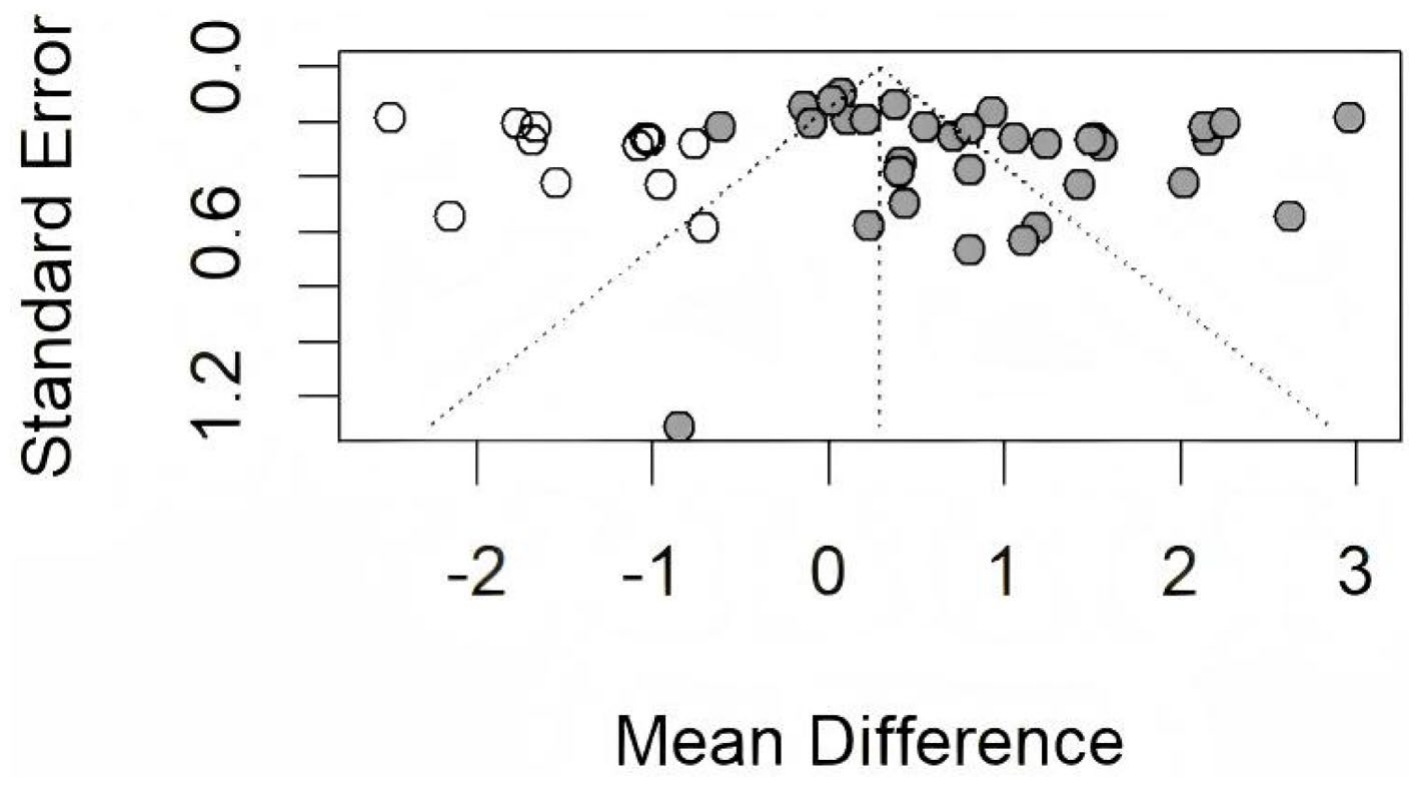
Funnel plot of HbA1c.

**Figure 10:**
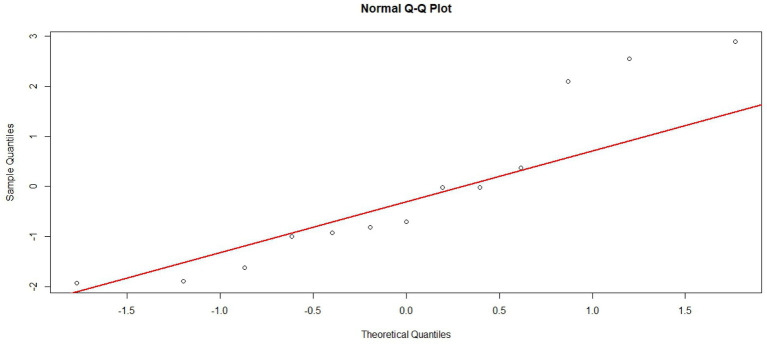
Normal Q-Q plot of the residuals from the random-effects meta-analysis for FINS.

To assess potential publication bias, funnel plots were generated and their symmetry was statistically evaluated using Egger’s test. The results indicated no significant publication bias for FPG (*p* = 0.4262), FINS (*p* = 0.1544), or HOMA-IR (*p* = 0.2164). However, Egger’s test indicated potential publication bias for HbA1c (*p* = 0.0382 < 0.05). A trim-and-fill analysis was subsequently performed to adjust for this bias, which imputed several hypothetical studies to achieve funnel plot symmetry. The adjusted effect size for HbA1c was MD = 0.29 (95% CI [0.09, 0.67]). This confirms that the observed bias did not materially alter the study’s conclusions. Funnel plots are presented in Figure 8, and the trim-and-fill adjusted plot for HbA1c is shown in Figure 9. Furthermore, to ensure the validity of the random-effects model, the normality of residuals was assessed using Q-Q plots (Figure 10) and the Shapiro–Wilk test. For FINS, the residuals followed a normal distribution (*W* = 0.881, *p* = 0.074), and the Q-Q plot showed no significant deviation from the reference line, suggesting that the distributional assumptions for the meta-analysis were generally acceptable, though the inferences regarding FINS should be interpreted with appropriate caution given the marginal *p*-value and observed tail distribution.

## 4 Discussion

The main findings of this systematic review and meta-analysis can be summarized as follows: (1) elevated protein expression level of HIF-1α in peripheral blood was significantly and positively associated with FPG (MD = 1.13, 95% CI [0.59, 1.67], *I*^2^ = 96.9%), HbA1c (MD = 0.93, 95% CI [0.63, 1.24], *I*^2^ = 94.0%), FINS (MD = 1.13, 95% CI [0.24, 2.02], *I*^2^ = 97.8%), and HOMA-IR (MD = 1.40, 95% CI [0.62, 2.18], *I*^2^ = 98.8%). (2) Subgroup analyses suggested that country might be a source of heterogeneity for the association with FPG, whereas age and geographical region (North vs. South China) showed no significant influence. (3) Meta-regression did not find a significant moderating effect of age and disease duration on outcome indicators. (4) Sensitivity analysis confirmed the robustness of the results. No significant publication bias was detected for FPG, FINS, or HOMA-IR. However, potential publication bias was indicated for HbA1c. Nevertheless, the trim-and-fill method demonstrated that the overall conclusions remained unchanged after adjustment. Although some previous clinical studies reported correlations between protein expression level of HIF-1α in peripheral blood and glycemic parameters, the results were inconsistent, and most were limited by small sample sizes and failed to systematically explore sources of heterogeneity. Our study, by incorporating global multi-regional studies and performing quantitative synthesis, significantly enhances statistical power and is the first to identify potential effect differences at the country level, suggesting that factors such as socioeconomic status, diagnostic/therapeutic standards, or population genetic background might modulate the relationship between HIF-1α and glucose metabolism.

The substantial heterogeneity (*I*^2^ > 90%) observed in this meta-analysis necessitates a cautious interpretation of the pooled results. Beyond the geographic differences identified through subgroup analysis, several biological and clinical factors likely contribute to this variance. Specifically, circulating HIF-1α levels may be influenced by individual patient characteristics such as body mass index, gender, the presence of diabetic complications, and even the altitude of the study location. Furthermore, the use of glucose-lowering medications, particularly SGLT2 inhibitors which have been shown to modulate the HIF signaling pathway, represents a significant confounding variable that could not be fully accounted for in our dataset (14). Methodologically, the current lack of global standardization in protein detection remains a critical concern. Discrepancies in the analytical sensitivity of different ELISA kits, alongside variations in blood sample processing, such as the use of plasma versus serum, may introduce considerable measurement error across studies. Consequently, these multifaceted factors are all possible reasons for the high heterogeneity of research, and due to the limitations of inclusion in the study, it is difficult to avoid them.

HIF-1α is a key transcription factor that mediates cellular responses to hypoxia and contributes centrally to several core pathological processes in T2DM, including insulin resistance and β-cell dysfunction (15–17). Recent large-scale data integration studies have further confirmed that the aberrant HIF-1α signaling pathway serves as the primary transcription factor and biomarker in the mRNA expression profile of rat and human pancreatic islets during the progression of T2DM, emphasizing its role in promoting aberrant angiogenesis in the pancreatic islets (18, 19). Clinical evidence predominantly indicates significantly elevated levels of HIF-1α in the peripheral blood of person with diabetes. However, a consistent yet complex observation is that HIF-1α expression exhibits inconsistent, and at times opposing, trends across different tissues. Under diabetic hypoxic conditions, HIF-1α protein expression increases in pancreatic β-cells and frequently localizes to regions of apoptosis, suggesting its potential role as a marker of hypoxic injury (20). Studies in animal models and high-glucose culture systems further demonstrate upregulation of HIF-1α and its downstream targets, such as glucose transporter 1 and 3-phosphoinositide-dependent protein kinase 1, in β-cells, implying that hyperglycemia may promote HIF-1α activation (21, 22). In contrast, other reports indicate that high glucose can impair HIF-1α protein stability and transcriptional activity, thereby disrupting this signaling pathway (23). Notably, β-cell–specific HIF-1α knockout mice display heightened susceptibility to damage, supporting a context-dependent protective role of HIF-1α in these cells (23). In the diabetic kidney, most studies describe elevated HIF-1α expression, which promotes lipid accumulation, fibrosis, inflammation, and the progression of renal injury (24–26). Nevertheless, conditional knockout of HIF-1α or inhibition of its downstream pathways has been shown in some settings to exacerbate kidney damage, suggesting that HIF-1α may exert protective functions at specific disease stages or within particular microenvironments (27, 28). Overall, HIF-1α expression in diabetes is modulated by multiple factors, including hyperglycemia, hypoxia, inflammation, disease duration, and cell type. A consensus has not yet been established, and the dynamic regulation of this pathway warrants further investigation.

Based on these seemingly contradictory yet interrelated findings, we propose that elevated circulating HIF-1α in T2DM may not be a primary driver of pathogenesis, but rather a systemic indicator of chronic hypoxia and a compensatory response to prolonged metabolic dysregulation. Specifically, while increased systemic HIF-1α levels reflect the overall burden of hypoxic stress, the decreased expression observed in target tissues—such as the pancreas and kidneys—may signal the exhaustion of local adaptive mechanisms. Thus, dynamic shifts in HIF-1α may serve as a barometer for systemic adaptive capacity, representing a consequence of, rather than a cause for, metabolic failure. From a clinical perspective, these findings position circulating HIF-1α as a potential marker for metabolic health. Monitoring HIF-1α levels could assist clinicians in identifying patients at higher risk of systemic hypoxic stress before the onset of overt microvascular complications. Specifically, the strong correlation with HbA1c and HOMA-IR suggests that HIF-1α might reflect the “metabolic memory” of chronic hyperglycemia. If validated in longitudinal cohorts, HIF-1α could serve as a supplementary diagnostic tool to refine risk stratification, moving beyond traditional glucose metrics to capture the underlying cellular stress response.

Several limitations must be acknowledged. First, the cross-sectional nature of the included studies precludes any causal inferences regarding the relationship between HIF-1α and islet function or insulin resistance. Second, variations in sample sources and control group definitions likely contribute to the observed residual heterogeneity, despite our use of a random-effects model. Third, the lack of granular data on potential confounders—such as medication use, body mass index, and renal function—prevented their inclusion in our meta-regression. These factors are known to modulate both HIF-1α expression and insulin sensitivity. Future prospective cohorts or IPD meta-analyses are required to further clarify these causal pathways. Finally, regarding statistical assumptions, while the Shapiro–Wilk test for FINS yielded a *p*-value of 0.07392, the heavy-tailed distribution observed in the Q-Q plot (Figure 10) suggests a subtle departure from strict normality. This distribution pattern implies that the pooled estimates for FINS should be interpreted with caution, as it may affect the precision of the resulting statistical inferences.

This study provides the most comprehensive assessment to date of the association between circulating HIF-1α and metabolic parameters in T2DM. Our results suggest that HIF-1α may serve as a valuable biomarker for metabolic control and offers insights into the molecular interplay between β cell dysfunction and insulin resistance. Future research should prioritize longitudinal studies to track the relationship between dynamic HIF-1α levels and islet function, potentially informing personalized glycemic management and targeted therapeutic interventions.

## 5 Conclusion

This systematic review and meta-analysis identifies a significant association between elevated circulating HIF-1α levels and both pancreatic β-cell dysfunction and increased insulin resistance. These findings point toward the involvement of hypoxic signaling in the pathophysiology of T2DM. However, these results should be interpreted with caution given the substantial clinical heterogeneity and potential statistical deviations observed in certain outcomes. Future research should prioritize longitudinal studies to clarify the specific mechanistic pathways and explore whether modulating HIF-1α activity can preserve β-cell function and improve insulin sensitivity.
