# Supplementary material for: The role of peripheral blood HIF-1α in pancreatic β-cell dysfunction and insulin resistance among patients with type 2 diabetes: a systematic review and meta-analysis
Source: Front Nutr. 2026 Apr 10;13:1763090. doi: 10.3389/fnut.2026.1763090 (PMC13106360; doi:10.3389/fnut.2026.1763090)
Supplement: Supplementary file 2 [file Data_Sheet_2.PDF]

NIHR | National Institute for Health and Care Research

PROSPERO
International prospective register of systematic reviews

Association between hypoxia-inducible factor-1α (HIF-1α) expression and pancreatic β-cell dysfunction in patients with type 2 diabetes mellitus: a systematic review and meta-analysis

Yi Su, rui Peng, jiahong Zhang

Citation
Yi Su, rui Peng, jiahong Zhang. Association between hypoxia-inducible factor-1α (HIF-1α) expression and pancreatic β-cell dysfunction in patients with type 2 diabetes mellitus: a systematic review and meta-analysis. PROSPERO 2025 CRD420251118501. Available from https://www.crd.york.ac.uk/PROSPERO/view/CRD420251118501.

REVIEW TITLE AND BASIC DETAILS

Review title

Association between hypoxia-inducible factor-1α (HIF-1α) expression and pancreatic β-cell dysfunction in patients with type 2 diabetes mellitus: a systematic review and meta-analysis

Condition or domain being studied

Hypoxia; Type 2 Diabetes Mellitus; C-peptide Level

Rationale for the review

One of the core pathologies of type 2 diabetes mellitus (T2DM) is the progressive failure of islet β cell function, which is characterized by insufficient or relative lack of insulin secretion. Recent studies have found that hypoxia inducible factor-1 α (HIF-1 α) plays a key regulatory role in glucose and lipid metabolism and oxidative stress signaling pathway, and its expression level is significantly increased in islets, peripheral blood or serum of T2DM patients. However, the existing research sample size is small, detection methods and population heterogeneity are strong, and the quantitative relationship between HIF-1 α elevation and β - cell failure is not clear. This systematic review and meta-analysis aimed to integrate global evidence to clarify the correlation between HIF-1 α expression and β - cell dysfunction in T2DM patients.-1 α) is a key transcription factor for cells to respond to hypoxia, playing an important role in local hypoxia and metabolic stress in the pancreas. It has been found that hyperglycemia in the environment of diabetes can inhibit the stability and activity of HIF-1 α, and damage the hypoxic adaptive response. In human pancreatic

islets, the expression level of HIF-1 α in T2DM patients was significantly lower than that in non diabetes patients, suggesting that HIF-1 α signal suppression may be one of the mechanisms of pancreatic islet dysfunction in T2DM.

Review objectives

How is the different expression levels of HIF-1α associated with pancreatic β -cell failure in patients with type 2 diabetes?

Keywords

Type 2 diabetes mellitus; Hypoxia-inducible factor 1α; Islet function

Country

China

ELIGIBILITY CRITERIA

Population

Included

Adult patients diagnosed with type 2 diabetes mellitus, regardless of the duration.(as diagnosed using any recognised diagnostic criteria).

Excluded

Study on non-type 2 diabetes mellitus (such as type 1 diabetes mellitus or gestational diabetes mellitus, etc.)

Intervention(s) or exposure(s)

Included

Clinical Support; Biopsy

Comparator(s) or control(s)

Included

PICO tags selected: Referral To Health Visitor; Expression

Study design

Only nonrandomized study types will be included.

Context

The included study needs to be an observational study carried out in the hospital, and the subjects are adults ⩾ 18 years old and diagnosed with type 2 diabetes mellitus (ada/who standard). The study must report the expression level of HIF-1 α (detection of pancreatic islet tissue, peripheral blood or serum), and provide β cell function indicators (HOMA-B, C-peptide or β cell apoptosis rate, etc.). Research sites are unlimited, but only for human research, excluding animals, intervention tests, conference summaries or reviews.

TIMELINE OF THE REVIEW

Date of first submission to PROSPERO

03 August 2025

Review timeline
Start date: 28 June 2025. End date: 31 March 2026.

Date of registration in PROSPERO
03 August 2025

AVAILABILITY OF FULL PROTOCOL

Availability of full protocol
A full protocol has not been written.

SEARCHING AND SCREENING

Search for unpublished studies
Only published studies will be sought.

Main bibliographic databases that will be searched
The main databases to be searched are *CENTRAL - Cochrane Central Register of Controlled Trials*, *CLIB - The Cochrane Library*, *Embase - Embase via Ovid*, *Embase.com*, *MEDLINE*, *PubMed*, *SCI - Science Citation Index* and *SSCI - Social Science Citation Index*.

Search language restrictions
The review will only include studies published in English and Chinese.

Search date restrictions
There are no search date restrictions.

Other methods of identifying studies
Other studies will be identified by: *contacting authors or experts*.

Link to search strategy
A full search strategy is not available.

Selection process
Studies will be screened independently by at least two people (or person/machine combination) with a process to resolve differences.

Other relevant information about searching and screening
None

DATA COLLECTION PROCESS

Data extraction from published articles and reports
Data will be extracted independently by at least two people (or person/machine combination) with a process to resolve differences.

Authors will be asked to provide any required data not available in published reports.

Study risk of bias or quality assessment
Risk of bias will be assessed using: *Newcastle-Ottawa*

Data will be assessed independently by at least two people (or person/machine combination) with a process to resolve differences.

Additional information will be sought from study investigators if required information is unclear or unavailable in the study publications/reports.

Reporting bias assessment

Risk of bias due to missing results will not be assessed

Certainty assessment

Calculate the effect size (e.g., relative risk - RR, odds ratio - OR, mean difference - MD) and its 95% confidence interval (95% CI) for each included study. Narrower CIs indicate higher certainty in the effect size estimate. If the CI includes the null value (e.g., RR or OR = 1, MD = 0), it shows statistical non - support for the effect, reflecting evidence uncertainty.

OUTCOMES TO BE ANALYSED

Main outcomes

HOMA-B , fasting C-peptide level

Additional outcomes

Proportion of insulin therapy needs

PLANNED DATA SYNTHESIS

Strategy for data synthesis

The random effect dersimonian Laird model (Hartung Knapp correction) was used to combine the standardized mean difference (SMD) or risk ratio (RR) to quantify the correlation between HIF-1 α and β cell function; The heterogeneity was evaluated by I^2 and Q tests, and the influence of sample source, course of disease, BMI and other confounding factors was explored by subgroup analysis; Further sensitivity analysis was performed by eliminating articles one by one, and funnel egger test was used to determine whether the included studies had publication bias; All analyses were completed in R.4.3.1, and the results strictly followed the PRISMA 2020 reporting specification.

CURRENT REVIEW STAGE

Stage of the review at this submission

Review stage	Started	Completed
Pilot work	✓	✓
Formal searching/study identification	✓	✓
Screening search results against inclusion criteria		
Data extraction or receipt of IPD		
Risk of bias/quality assessment		
Data synthesis		

Review status
The review is currently planned or ongoing.

Publication of review results
Results of the review will be published.

REVIEW AFFILIATION, FUNDING AND PEER REVIEW

Review team members
Ms Yi Su (review guarantor and contact) Chengdu University of Traditional Chinese Medicine. China.

No conflict of interest declared.

Mr rui Peng. Chengdu University of Traditional Chinese Medicine. China.

No conflict of interest declared.

Miss jiahong Zhang. Chengdu University of Traditional Chinese Medicine. China.

No conflict of interest declared.

Named contact
Ms Yi Su (1426965520@qq.com). Chengdu University of Traditional Chinese Medicine. China.

Review affiliation
Chengdu University of Traditional Chinese Medicine

Funding source
Review has no specific/external funding but is supported by guarantor/review team (non-commercial) institutions.

Peer review
There has been no peer review of this planned review.

ADDITIONAL INFORMATION

Review conflict of interest
Declared individual interests are recorded under team member details.. No additional interests are recorded for this review.

Medical Subject Headings
Diabetes Mellitus, Type 2; Hypoxia-Inducible Factor 1, alpha Subunit

SIMILAR REVIEWS

Check for similar records already in PROSPERO
PROSPERO identified a number of existing PROSPERO records that were similar to this one (last check made on 3 August 2025). These are shown below along with the reasons given by that the review team for the reviews being different and/or proceeding.

- Prognostic and clinicopathological significance of hypoxia-inducible factors 1α and 2α in hepatocarcinoma: A systematic review with meta-analysis. [published 14 July 2020] [CRD42020191977]. The review was judged **not to be similar**
- The prognostic significance of HIF-1α expression in patients with glioma: A meta-analysis [published 4 February 2021] [CRD42021229104]. The review was judged **not to be similar**
- Expression of HIF-1α in Ameloblastoma: A Systematic Review [published 9 September 2022] [CRD42022356660]. The review was judged **not to be similar**

PROSPERO version history

- Version 1.0, published 03 Aug 2025

Disclaimer

The content of this record displays the information provided by the review team. PROSPERO does not peer review registration records or endorse their content.

PROSPERO accepts and posts the information provided in good faith; responsibility for record content rests with the review team. The guarantor for this record has affirmed that the information provided is truthful and that they understand that deliberate provision of inaccurate information may be construed as scientific misconduct.

PROSPERO does not accept any liability for the content provided in this record or for its use. Readers use the information provided in this record at their own risk.

Any enquiries about the record should be referred to the named review contact
