# Supplementary material for: The role of peripheral blood HIF-1α in pancreatic β-cell dysfunction and insulin resistance among patients with type 2 diabetes: a systematic review and meta-analysis
Source: Front Nutr. 2026 Apr 10;13:1763090. doi: 10.3389/fnut.2026.1763090 (PMC13106360; doi:10.3389/fnut.2026.1763090)
Supplement: Supplementary file 3 [file Data_Sheet_3.PDF]

Pubmed

Search	Query	Results	Date
#1	"diabetes mellitus, type 2"[MeSH Terms]	191,488	2025/7/28
#2	"type II diabetes mellitus"[Title/Abstract] OR "type II diabetes"[Title/Abstract] OR "type 2 diabetes*"[Title/Abstract] OR "TIIDM"[Title/Abstract] OR "T2DM"[Title/Abstract] OR "Stable Diabetes Mellitus"[Title/Abstract] OR "noninsulin dependent diabetes*"[Title/Abstract] OR "non insulin dependent diabetes*"[Title/Abstract] OR "Non Insulin Dependent Diabetes Mellitus"[Title/Abstract] OR "NIDDM"[Title/Abstract] OR "MODY"[Title/Abstract] OR "maturity onset diabetes*"[Title/Abstract] OR "Ketosis Resistant Diabetes Mellitus"[Title/Abstract] OR "insulin independent diabetes*"[Title/Abstract] OR "dm 2"[Title/Abstract] OR "diabetes type 2"[Title/Abstract] OR "diabetes maturity onset"[Title/Abstract] OR "diabetes adult onset"[Title/Abstract] OR "diabetes type II"[Title/Abstract] OR "diabetes type 2"[Title/Abstract] OR "diabetes mellitus type ii"[Title/Abstract] OR "diabetes mellitus stable"[Title/Abstract] OR "diabetes mellitus slow onset"[Title/Abstract] OR "diabetes mellitus noninsulin dependent"[Title/Abstract] OR "diabetes mellitus non insulin dependent"[Title/Abstract] OR "diabetes mellitus maturity onset"[Title/Abstract] OR "diabetes mellitus adult onset"[Title/Abstract] OR "diabetes mellitus type ii"[Title/Abstract] OR "diabetes mellitus type 2"[Title/Abstract] OR "adult onset diabetes*"[Title/Abstract]	227,977	2025/7/28
#3	"hypoxia inducible factor 1, alpha subunit"[MeSH Terms]	18,767	2025/7/28
#4	"hypoxia inducible factor 1alpha"[Title/Abstract] OR "hypoxia inducible factor 1 alpha subunit"[Title/Abstract] OR "ARNT interacting protein"[Title/Abstract]	9,952	2025/7/28
#5	("diabetes mellitus, type 2"[MeSH Terms] OR ("type II diabetes mellitus"[Title/Abstract] OR "type II diabetes"[Title/Abstract] OR "type 2	185	2025/7/28

	diabetes*"[Title/Abstract] OR "TIIDM"[Title/Abstract] OR "T2DM"[Title/Abstract] OR "Stable Diabetes Mellitus"[Title/Abstract] OR "noninsulin dependent diabetes*"[Title/Abstract] OR "non insulin dependent diabetes*"[Title/Abstract] OR "Non Insulin Dependent Diabetes Mellitus"[Title/Abstract] OR "NIDDM"[Title/Abstract] OR "MODY"[Title/Abstract] OR "maturity onset diabetes*"[Title/Abstract] OR "Ketosis Resistant Diabetes Mellitus"[Title/Abstract] OR "insulin independent diabetes*"[Title/Abstract] OR "dm 2"[Title/Abstract] OR "diabetes type 2"[Title/Abstract] OR "diabetes maturity onset"[Title/Abstract] OR "diabetes adult onset"[Title/Abstract] OR "diabetes type II"[Title/Abstract] OR "diabetes type 2"[Title/Abstract] OR "diabetes mellitus type ii"[Title/Abstract] OR "diabetes mellitus stable"[Title/Abstract] OR "diabetes mellitus slow onset"[Title/Abstract] OR "diabetes mellitus noninsulin dependent"[Title/Abstract] OR "diabetes mellitus non insulin dependent"[Title/Abstract] OR "diabetes mellitus maturity onset"[Title/Abstract] OR "diabetes mellitus adult onset"[Title/Abstract] OR "diabetes mellitus type ii"[Title/Abstract] OR "diabetes mellitus type 2"[Title/Abstract] OR "adult onset diabetes*"[Title/Abstract])) AND ("hypoxia inducible factor 1, alpha subunit"[MeSH Terms] OR ("hypoxia inducible factor 1alpha"[Title/Abstract] OR "hypoxia inducible factor 1 alpha subunit"[Title/Abstract] OR "ARNT interacting protein"[Title/Abstract]))		

Embase

Search	Query	Results	Date
#1	'non insulin dependent diabetes mellitus'/exp	426,540	2025/7/28
#2	'type ii diabetes mellitus':ab,ti,kw OR 'type ii diabetes':ab,ti,kw OR 'type 2 insulin independent diabetes':ab,ti,kw OR 'type 2 diabetes*':ab,ti,kw OR 'tiidm':ab,ti,kw OR 't2dm':ab,ti,kw OR 'stable diabetes mellitus':ab,ti,kw OR 'slow onset diabetes mellitus':ab,ti,kw OR 'noninsulin dependent type 2 diabetes mellitus':ab,ti,kw OR 'noninsulin dependent diabetes*':ab,ti,kw OR 'non insulin dependent diabetes*':ab,ti,kw OR 'non insulin dependent diabetes mellitus':ab,ti,kw OR 'niddm':ab,ti,kw OR 'mody':ab,ti,kw OR 'maturity onset diabetes*':ab,ti,kw OR 'ketosis resistant diabetes mellitus':ab,ti,kw OR 'insulin independent diabetes*':ab,ti,kw OR 'dm 2':ab,ti,kw OR 'diabetes, type 2':ab,ti,kw OR 'diabetes, maturity onset':ab,ti,kw OR 'diabetes, adult onset':ab,ti,kw OR 'diabetes type ii':ab,ti,kw OR 'diabetes type 2':ab,ti,kw OR 'diabetes mellitus, type ii':ab,ti,kw OR 'diabetes mellitus, stable':ab,ti,kw OR 'diabetes mellitus, slow onset':ab,ti,kw OR 'diabetes mellitus, noninsulin dependent':ab,ti,kw OR 'diabetes mellitus, non insulin dependent':ab,ti,kw OR 'diabetes mellitus, maturity onset':ab,ti,kw OR 'diabetes mellitus, ketosis resistant':ab,ti,kw OR 'diabetes mellitus, adult onset':ab,ti,kw OR 'diabetes mellitus type ii':ab,ti,kw OR 'diabetes mellitus type 2':ab,ti,kw OR 'adult onset diabetes*':ab,ti,kw	365,483	2025/7/28
#3	'hypoxia inducible factor 1alpha'/exp	40,878	2025/7/28
#4	'member of pas protein 1':ab,ti,kw OR 'hypoxia inducible factor 1alpha':ab,ti,kw OR 'hypoxia inducible factor 1 alpha subunit':ab,ti,kw OR 'arnt interacting protein':ab,ti,kw	1,609	2025/7/28
#5	#1 OR #2	492,509	2025/7/28
#6	#3 OR #4	41,098	2025/7/28
#7	#5 AND #6	621	2025/7/28

Cochrane library

Search	Query	Results	Date
#1	MeSH descriptor: [Diabetes Mellitus, Type 2] explode all trees	26788	2025-07-29
#2	('type II diabetes mellitus' OR 'type II diabetes' OR 'type 2 insulin independent diabetes' OR 'Type 2 Diabetes*' OR 'TIIDM' OR 'T2DM' OR 'Stable Diabetes Mellitus' OR 'Slow Onset Diabetes Mellitus' OR 'noninsulin dependent type 2 diabetes mellitus' OR 'noninsulin dependent diabetes*' OR 'non insulin dependent diabetes*' OR 'Non Insulin Dependent Diabetes Mellitus' OR 'NIDDM' OR 'MODY' OR 'Maturity Onset Diabetes*' OR 'Ketosis Resistant Diabetes Mellitus' OR 'insulin independent diabetes*' OR 'dm 2' OR 'Diabetes, Type 2' OR 'Diabetes, Maturity Onset' OR 'diabetes, adult onset' OR 'diabetes type II' OR 'diabetes type 2' OR 'Diabetes Mellitus, Type II' OR 'Diabetes Mellitus, Stable' OR 'Diabetes Mellitus, Slow Onset' OR 'Diabetes Mellitus, Noninsulin Dependent' OR 'Diabetes Mellitus, Non Insulin Dependent' OR 'Diabetes Mellitus, Maturity Onset' OR 'Diabetes Mellitus, Ketosis Resistant' OR 'Diabetes Mellitus, Adult Onset' OR 'diabetes mellitus type ii' OR 'diabetes mellitus type 2' OR 'adult onset diabetes*'):ab,ti,kw	75306	2025-07-29
#3	MeSH descriptor: [Hypoxia-Inducible Factor 1, alpha Subunit] explode all trees	100	2025-07-29
#4	('member of PAS protein 1' OR 'hypoxia inducible factor 1alpha' OR 'hypoxia inducible factor 1 alpha subunit' OR 'ARNT interacting protein'):ab,ti,kw	125	2025-07-29
#5	(#1 OR #2)　AND (#3 OR #4)	5	2025-07-29

Web of Science

Search	Query	Results	Date
#1	TS=("type II diabetes mellitus" OR "type II diabetes" OR "type 2 insulin independent diabetes" OR "Type 2 Diabetes*" OR "tiide" OR "T2DM" OR "Stable Diabetes Mellitus" OR "Slow Onset Diabetes Mellitus" OR "noninsulin dependent type 2 diabetes mellitus" OR "noninsulin dependent diabetes*" OR "non insulin dependent diabetes*" OR "Non Insulin Dependent Diabetes Mellitus" OR "NIDDM" OR "MODY" OR "Maturity Onset Diabetes*" OR "Ketosis Resistant Diabetes Mellitus" OR "insulin independent diabetes*" OR "dm 2" OR "Diabetes, Type 2" OR "Diabetes, Maturity Onset" OR "diabetes, adult onset" OR "diabetes type II" OR "diabetes type 2" OR "Diabetes Mellitus, Type II" OR "Diabetes Mellitus, Stable" OR "Diabetes Mellitus, Slow Onset" OR "Diabetes Mellitus, Noninsulin Dependent" OR "Diabetes Mellitus, Non Insulin Dependent" OR "Diabetes Mellitus, Maturity Onset" OR "Diabetes Mellitus, Ketosis Resistant" OR "Diabetes Mellitus, Adult Onset" OR "diabetes mellitus type ii" OR "diabetes mellitus type 2" OR "adult onset diabetes*" OR "non insulin dependent diabetes mellitus" OR "Diabetes Mellitus, Type 2")	372,932	2025-07-29
#2	TS=("member of PAS protein 1" OR "hypoxia inducible factor 1alpha" OR "hypoxia inducible factor 1 alpha subunit" OR "ARNT interacting protein" OR "Hypoxia-Inducible Factor 1, alpha Subunit" OR "hypoxia inducible factor 1alpha")	23,158	2025-07-29
#3	#1 AND #2	197	2025-07-29

VIP

Search	Query	Results	Date
#1	题名或关键词=2 型糖尿病+diabetes melitus type 2+noninsulin- dependent diabetes mellitus+type2 diabetes+type 2 diabetes mellitus+type 2 diabetic+type ii diabetes+type ii diabetes mellitus+成人发病型糖尿病+糖尿病 2 型+第二型糖尿病+非胰岛素依赖性糖尿病+非胰岛素依赖型糖尿病+二型糖尿病 AND 题名或关键词=缺氧诱导因子 1,α 亚基+HIF-1α AND 期刊范围:北大核心期刊,EI 来源期刊,SCIE 期刊,CAS 来源期刊	30	2025/7/28

Wanfang

Search	Query	Results	Date
#1	题名或关键词:(2 型糖尿病 OR 成人发病型糖尿病 OR 糖尿病 2 型 OR 第二型糖尿病 OR 非胰岛素依赖性糖尿病 OR 非胰岛素依赖型糖尿病 OR 二型糖尿病) and 题名或关键词:(缺氧诱导因子 1,α 亚基 OR HIF-1α)	42	2025-07-29

CNKI

Search	Query	Results	Date
#1	TKA = ('2 型糖尿病'+'diabetes melitus type 2'+'noninsulin- dependent diabetes mellitus'+'type2 diabetes'+'type 2 diabetes mellitus'+'type 2 diabetic'+'type ii diabetes'+'type ii diabetes mellitus'+'成人发病型糖尿病'+'糖尿病 2 型'+'第二型糖尿病'+'非胰岛素依赖性糖尿病'+'非胰岛素依赖型糖尿病'+'二型糖尿病') AND TKA = ('缺氧诱导因子 1,α 亚基'+'HIF-1α')	105	2025-07-29

Sinomed

Search	Query	Results	Date
#1	"缺氧诱导因子 1,α 亚基"[全部字段:智能] OR "HIF-1α"[全部字段:智能]	9827	2025-07-29
#2	"2 型糖尿病"[全部字段:智能] OR "成人发病型糖尿病"[全部字段:智能] OR "糖尿病 2 型"[全部字段:智能] OR "第二型糖尿病"[全部字段:智能] OR "非胰岛素依赖性糖尿病"[全部字段:智能] OR "非胰岛素依赖型糖尿病"[全部字段:智能] OR "二型糖尿病"[全部字段:智能]	146408	2025-07-29
#3	(#2) AND (#1)	96	2025-07-29
