# Supplementary material for: The role of peripheral blood HIF-1α in pancreatic β-cell dysfunction and insulin resistance among patients with type 2 diabetes: a systematic review and meta-analysis
Source: Front Nutr. 2026 Apr 10;13:1763090. doi: 10.3389/fnut.2026.1763090 (PMC13106360; doi:10.3389/fnut.2026.1763090)
Supplement: Supplementary file 4 [file Data_Sheet_4.PDF]

AHRQ Cross-Sectional Study Evaluation Criteria

	Study	Shao et al.	Shao et al.	Che et al.	Tang et al.	Shen et al.	E et al.	Zhang et al.	Liu et al.	Li et al.	Li et al.	Lu et al.	Lu et al.	Shao et al.	Shao et al.	Song et al.	Song et al.	Sun et al.	Xu et al.
1	Define the source of information (survey, record review).	Yes	Yes	Yes	Yes	Yes	Yes	Yes	Yes	Yes	Yes	Yes	Yes	Yes	Yes	Yes	Yes	Yes	Yes
2	List inclusion and exclusion criteria for exposed and unexposed subjects (cases and controls) or refer to previous publications.	Yes	Yes	Yes	Yes	Yes	Yes	Yes	Yes	Yes	Yes	Yes	Yes	Yes	Yes	Yes	Yes	Yes	Yes
3	Indicate time period used for identifying patients.	Yes	Yes	Yes	Yes	Yes	Yes	Yes	Yes	No	Yes	No	No	Yes	Yes	Yes	Yes	Yes	Yes
4	Indicate whether or not subjects were consecutive if not population-based.	No	No	No	No	No	No	No	No	No	No	No	No	No	No	No	No	No	No
5	Indicate if evaluators of subjective components of study were masked to other aspects of the participants.	Yes	Yes	Yes	Yes	Yes	Yes	Yes	Yes	Yes	Yes	Yes	Yes	Yes	Yes	Yes	Yes	Yes	Yes
6	Describe any assessments undertaken for quality assurance purposes (e.g.,test/retest of primary outcome	Yes	No	Yes	No	Yes	No	Yes	No	No	Yes	Yes	Yes	No	No	No	Yes	No	No
7	Explain any patient exclusions from analysis.	Yes	Yes	Yes	Yes	Yes	Yes	No	No	No	Yes	Yes	Yes	Yes	Yes	Yes	Yes	Yes	Yes
8	Describe how confounding was assessed and/or controlled.	Yes	Yes	Yes	Yes	No	Yes	Yes	Yes	Yes	Yes	Yes	No	Yes	Yes	No	No	Yes	No
9	If applicable, explain how missing data were handled in the analysis.	Yes	Yes	Yes	Yes	Yes	Yes	No	No	No	Yes	Yes	Yes	Yes	Yes	Yes	Yes	Yes	Yes
10	Summarize patient response rates and completeness of data collection.	Yes	Yes	Yes	Yes	Yes	Yes	Yes	Yes	Yes	Yes	Yes	Yes	Yes	Yes	Yes	Yes	Yes	Yes
11	Clarify what follow-up, if any, was expected and the percentage of patients for which incomplete data or follow-up was obtained.	No	No	No	No	No	No	No	No	No	No	No	No	No	No	No	No	No	No
	**Total score**	9	8	9	8	8	8	7	6	5	9	8	7	8	8	7	8	8	7

	Study	Xu et al.	Xue et al.	Zhang et al.	Zhu et al.	Shan et al.	Tian et al.	Su et al.	Shao et al.	Shao et al.	Sayed et al.	Rusdiana et al.	Lv et al.	Li et al.	Jiang et al.	GHARIB et al.	Gharib et al.	Gaonkar et al.	Cuore et al.
1	Define the source of information (survey, record review).	Yes	Yes	Yes	Yes	Yes	Yes	Yes	Yes	Yes	Yes	Yes	Yes	Yes	Yes	Yes	Yes	Yes	Yes
2	List inclusion and exclusion criteria for exposed and unexposed subjects (cases and controls) or refer to previous publications.	Yes	Yes	Yes	Yes	Yes	Yes	Yes	Yes	Yes	Yes	Yes	Yes	Yes	Yes	Yes	Yes	Yes	Yes
3	Indicate time period used for identifying patients.	Yes	Yes	Yes	Yes	Yes	Yes	Yes	Yes	Yes	No	Yes	Yes	Yes	No	Yes	Yes	No	Yes
4	Indicate whether or not subjects were consecutive if not population-based.	No	No	No	No	No	No	No	Yes	Yes	No	Yes	Yes	Yes	No	No	No	No	No
5	Indicate if evaluators of subjective components of study were masked to other aspects of the participants.	Yes	Yes	Yes	Yes	Yes	Yes	Yes	Yes	Yes	Yes	Yes	Yes	Yes	Yes	Yes	Yes	Yes	Yes
6	Describe any assessments undertaken for quality assurance purposes (e.g.,test/retest of primary outcome	No	No	No	No	No	No	Yes	Yes	Yes	Yes	Yes	Yes	Yes	No	Yes	No	Yes	Yes
7	Explain any patient exclusions from analysis.	Yes	Yes	Yes	Yes	Yes	Yes	Yes	Yes	Yes	Yes	Yes	Yes	Yes	Yes	Yes	Yes	Yes	Yes
8	Describe how confounding was assessed and/or controlled.	Yes	No	Yes	Yes	Yes	Yes	Yes	Yes	Yes	Yes	Yes	Yes	Yes	No	Yes	Yes	Yes	Yes
9	If applicable, explain how missing data were handled in the analysis.	Yes	Yes	Yes	Yes	Yes	Yes	Yes	Yes	Yes	Yes	Yes	Yes	Yes	Yes	Yes	Yes	Yes	Yes
10	Summarize patient response rates and completeness of data collection.	Yes	Yes	Yes	Yes	Yes	Yes	Yes	Yes	Yes	Yes	Yes	Yes	Yes	Yes	Yes	Yes	Yes	Yes
11	Clarify what follow-up, if any, was expected and the percentage of patients for which incomplete data or follow-up was obtained.	No	No	No	No	No	No	No	No	No	No	No	No	No	No	No	No	No	No
	**Total score**	8	7	8	8	8	8	9	10	10	8	10	10	10	6	9	8	8	9
